# Calixarbutin: A Novel Calixarene-based Potential Water-soluble Anti-tyrosinase Agent with High Anti-melanoma Activity

**DOI:** 10.22037/ijpr.2020.112506.13798

**Published:** 2020

**Authors:** Jalal Ghaffarzadeh, Fazel Nasuhi Pur

**Affiliations:** a *Deputy of Food and Drug Administration, Urmia University of Medical Sciences, Urmia, Iran. *; b *Health Technology Incubation Center, Urmia University of Medical Sciences, Urmia, Iran.*

**Keywords:** Anti-tyrosinase, Anti-melanoma, Arbutin, Calixarene, Cluster

## Abstract

Since melanocytes are the origin of melanoma and some skin disorders such as melasma, they are important cells from the perspective of medicinal chemistry. Therefore, a medication that can simultaneously overcome these diseases will be a successful potential therapeutic agent. Arbutin with phenolic structure is a powerful natural anti-tyrosinase agent. Hence, the phenolic structure of this drug, prompted us to design its novel calix [4]arene-based cluster. Therefore, the present study reports the synthesis and *in-vitro* bio-activities of cyclic tetramer of arbutin in comparison to its simple drug unit as the reference medication. The *in-vitro* biological results showed amplified anti-tyrosinase (6-fold) and anti-melanoma (27-fold) activities, in addition to more aqueous solubility (8-fold) for this cluster in relation to arbutin. Therefore, compared to arbutin, more bioactive cluster can be considered as a novel water-soluble melanogenesis inhibitor with high anti-melanoma activity.

## Introduction

Among the strategies for designing new drugs, one of the most effective strategies is the use of established therapeutic drugs with important advantages such as\ time and cost saving, safety/tolerability, and availability of medications (1). Specially, clustering of the established therapeutic drug agents on a suitable molecular scaffold, can be one of the successful strategies that leads to the formation of cluster structures of the certain drugs. These drug clusters can have enhanced bio-activities (via impacting and synergizing effects of the drug units) than the related drug units (1). 

Since skin melanocytes are the origin of melanoma and some skin disorders such as melasma for these cells, a drug will be successful that will affect both of these diseases, simultaneously (2, 3). 

**Scheme 1:**
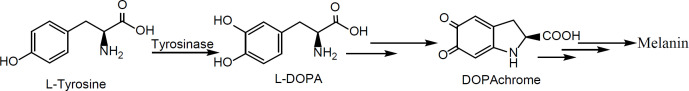
The biosynthetic pathway of melanin pigment

Tyrosinase is a key enzyme in the first oxidation step of the melanin biosynthesis process in melanocytes (Scheme 1) (2, 3). So, by replacing its two natural substrates (L-tyrosine and/or L-dihydroxyphenylalanine (L-DOPA)) with an alternative synthetic substrate (inhibitor), it will be possible to inhibit melanogenesis.

Arbutin is a natural β-D-glucopyranoside of hydroquinone with tyrosinase inhibitory effect and demonstrates excellent safety without any toxicity and irritation (4, 5). Arbutin has diverse functions, including depigmenting activity for treatment of melasma and disorders of hyperpigmentation and anticancer activities (5-7). The depigmenting mechanism of arbutin in humans involves the inhibition of melanosomal tyrosinase activity, rather than the suppression of the expression or synthesis of the enzyme (7).

On the other hand, calixarenes as cyclic oligomers of phenols are suitable structures (without any notable *in-vivo* toxicity and immune responses) for designing and developing of new drugs via clustering of single phenolic drug units such as arbutin (8-14).

So, in our chained studies for the synthesis of new calixdrugs, calixarene-based clusters of established therapeutic drug agents, in the present study, the authors introduce novel cyclic tetramer of arbutin, based on calixarene scaffold as its cluster with potential anti-tyrosinase and anti-melanoma activities (1). Based on calixarenes’ nomenclature, the chalice-shaped cluster of arbutin was innovatively named calixarbutin (1).

In summary, due to the phenolic moiety of the arbutin structure and considering the fact that, calixarenes are the cyclic oligomer of phenols, by performing a series of chemical reactions on the calix [4]arene, the arbutin cluster based on the calixarene scaffold can be obtained. On the other hand, due to the clustering effect of cluster structures, which results from their multivalency and the synergistic effects of phenolic units, in principle, calixarbutin should has more bioactivities than arbutin by default. Therefore, to evaluate this clustering effect, calixarbutin was compared with arbutin in two anti-tyrosinase and anti-melanoma tests. 

## Experimental

*General*

The melting points of all compounds were determined on a Philip Harris C4954718 apparatus. The optically active samples were analyzed by EHARTNACK apparatus at 20 °C. The ^1^H-NMR (400 MHz) and ^13^C-NMR (100 MHz) measurements were performed on a Bruker AM-400 spectrometer in CDCl_3_, and D_2_O using TMS as the internal reference. Mass spectra were recorded on a JEOL-JMS 600 (FAB-MS) instrument. All chemicals were purchased from Merck and Aldrich (Tehran, Iran) and used as received. A375 cell line (CRL-1619) was purchased from the American Tissue Culture Collection (ATCC).

*Synthetic procedures*

Compound **1 **with 1,3-alternative conformation was prepared by the previously reported method as white crystals (13). 2,3,4,6-Tetraacetyl-α-D-glucopyranosyl bromide was prepared according to the published method (15).

*1,3-alternate 5,11,17,23-Tetraacetyl-tetrakis(tetra-acetyl-β-D-glucopyranosyloxy)calix[4]arene (2)*

To a stirred mixture of compound **1** (2.37 g, 4 mmol) and K_2_CO_3_ (4.35 g, 30 mmol) in acetone (50 mL) was added a solution of NaI (4.65 g, 30 mmol) and 2,3,4,6-tetra-O-acetyl-α-D-glucopyranosylbromide (12.33 g, 30 mmol) in acetone (30 mL). The reaction mixture was heated to reflux under N_2_ atmosphere for 6 h. Then it was cooled, filtered, and washed with fresh acetone (20 mL). After solvent evaporation, the residue was suspended in water (30 mL) at 60 °C and stirred for 2 h. Then the product was extracted into the dichloromethane (20 mL). By removing the solvent a solid was formed which on recrystallization from acetone furnished **2** as white crystals. 

Compound **2**: yield, 72%; m.p. 202-204 °C; [α]_D_^20^ = – 13° (c = 0.5, CH_2_Cl_2_); ^1^H-NMR (400 MHz; CDCl_3_; δ, ppm): 6.67 (s, 8H, ArH), 4.32 (d. 4H, J_1,2_ = 7.2 Hz; H-l), 4.02 (s, 8H, ArCH_2_Ar), 3.56-3.64 (m, 4H, H-4), 3.44-3.52 (m, 8H, H-6a,b), 3.26-3.34 (m, 12H, H-2,3,5), 2.54 (s, 12H, CH_3_), 2.12 (s, 12H, OAc), 2.09 (s, 12H, OAc), 2.07 (s, 12H, OAc), 1.99 (s, 12H, OAc); ^13^C-NMR (100 MHz; CDCl_3_; δ, ppm): 190.7 (C=O) 171.5-171.8 (4C=O), 153.4, 143.9, 128.8, 127.9, 101.7, 76.9, 76.9, 73.1, 69.5, 60.7, 36.8 (ArCH_2_Ar). 27.1 (CH_3_), 20.8-20.9 (4OAc); FAB-MS (m/z): 1913.52 (M+).

*1,3-alternate 5,11,17,23-Tetraacetoxy-tetrakis((tetra-acetyl-β-D-glucopyranosyloxy)calix[4]arene (3)*

A mixture of compound **2** (5.74 g, 3 mmol) and perbenzoic acid (3.45 g, 25 mmol) of in chloroform (40 mL) was allowed to stand for a week in the dark, with occasional shaking at room temperature. The reaction mixture was washed with 1 N Na_2_CO_3_, washed with water, and dried. The chloroform was evaporated under reduced pressure to give a solid. Recrystallization from acetone yielded compound **3 **as white powder. 

Compound **3**: yield, 81%; m.p. 192-194 °C; [α]_D_^20^ = – 6° (c = 0.5, CH_2_Cl_2_); ^1^H-NMR (400 MHz; CDCl_3_; δ, ppm): 6.82 (s, 8H, ArH), 4.36 (d. 4H, J_1,2_ = 7.2 Hz; H-l), 3.99 (s, 8H, ArCH_2_Ar), 3.58-3.67 (m, 4H, H-4), 3.40-3.49 (m, 8H, H-6a,b), 3.23-3.32 (m, 12H, H-2,3,5), 2.21 (s, 12H, CH_3_) 2.11 (s, 12H, OAc), 2.09 (s, 12H, OAc), 2.06 (s, 12H, OAc), 1.98 (s, 12H, OAc); ^13^C-NMR (100 MHz; CDCl_3_; δ, ppm): 171.4-171.6 (4C=O), 169.7 (C=O), 153.2, 141.9, 129.0, 128.2, 101.3, 77.2, 77.0, 72.9, 69.8, 60.7, 36.9 (ArCH_2_Ar). 20.7-20.9 (5OAc); FAB-MS (m/z): 1977.56 (M+).

*1,3-alternate 5,11,17,23-Tetrahydroxyl-tetrakis((β-D-glucopyranosyloxy)calix[4]arene (1,3-alt-Calixarbutin, 4)*

A solution of compound **3** (3.95 g, 2 mmol) in aqueous 10% NaOH solution of dioxane (60 mL) was refluxed under N_2_ for 2 h. After cooling, the solution was acidified with aqueous 1% HCl until the acidity of the solution reached pH 3. Most of the solvent was removed under reduced pressure to give a residue. To the residue was added 500 mL of water to yield a white precipitate. Then the precipitate was added a solution of 5% NaOMe/MeOH until the basicity of the solution reached pH 9.5~10. The reaction mixture was stirred under argon atmosphere for 5 h. Then the solvent methanol was evaporated to give a residue. Recrystallization from MeOH/H_2_O containing small amounts of HC1 obtained compound **4** as white powder. 

Compound **4**: yield, 90%; m.p. 222-224 °C; [α]_D_^20^ = – 30° (c = 0.5, MeOH); ^1^H-NMR (400 MHz; D_2_O; δ, ppm): 6.77 (s, 8H, ArH), 4.37 (d. 4H, J_1,2_ = 7.6 Hz; H-l), 4.06 (s, 8H, ArCH_2_Ar), 3.68-3.72 (m, 4H, H-4), 3.48-3.59 (m, 8H, H-6a,b), 3.24-3.35 (m, 12H, H-2,3,5); ^13^C-NMR (100 MHz; D_2_O; δ, ppm): 152.8, 150.9, 129.2, 128.1, 101.7, 76.9, 76.6, 73.4, 70.1, 61.2, 37.1 (ArCH_2_Ar); FAB-MS (m/z): 1137.34 (M+).

*Mushroom tyrosinase assay*

*In-vitro* mushroom tyrosinase inhibitory assay was performed using the DOPA-chrome method with some modifications (16). The reaction mixtures, consisting of L-DOPA (1 mM), and the inhibitor (concentration range 10–300 μM) in phosphate buffer (50 mM, pH 6.8), were preincubated at room temperature for 10 min. Mushroom tyrosinase (10 U/mL) solution dissolved in phosphate buffer (50 mM, pH 6.8) was then added, and the reaction mixtures were incubated for 30 min at room temperature in 96-well plates to determine IC_50 _values. The absorbance was measured at 492 nm using a spectrophotometric microplate reader.

*Cell proliferation assay*

Potential cytotoxicity was evaluated against an *in-vitro* panel of A375 human malignant melanoma cell line (CRL-1619, ATCC) using previously published method with some modifications (8). Compound **4** was pre-dissolved in water and arbutin was pre-dissolved in DMSO (5 mM for avoiding DMSO toxicity) and diluted with cell culture medium to six required concentrations (1, 2, 10, 20, 100, and 1000 μg/mL). All cells were cultured in RPMI-1640 supplemented with 10% fetal bovine serum (FBS), 4 mM glutamine, 100 IU/mL benzylpenicillin, and 100 μg/mL streptomycin at 37 °C in a humidified atmosphere of 5% CO_2_ for 24 h. The cells were seeded at a density of 5 × 10^4^ cells per well in 96-well microplates. After 48 h, the cells were treated with a serial concentration of the test compound. The cells were exposed to the drugs in microplates, which were incubated under tissue culture conditions for 72 h. The cell growth was assayed using the colorimetric MTT assay by measurement of the optical density at a wavelength of 450 nm by a microplate reader. The values are mean for data from at least three independent experiments with quadruplicate readings in each experiment.

## Results and Discussion

*Chemistry*

**Scheme 2:**
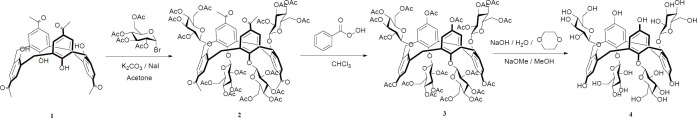
Synthetic route of calixarbutin (4).

Considering the structure of arbutin metabolites, the formation possibility of ortho-quinone in the calixarene-based structure should also be provided to lead the reduction of the tyrosinase enzyme (17). Therefore, this possibility is not present at the lower rim hydroxyl groups of the calixarene structure (due to the presence of methylene bridges in ortho position of hydroxyl groups), and so, the free hydroxyl group should be located at the upper rim of the calixarene structure to result in the ortho-quinone formation. Therefore, according to the above explanation, we designed the following synthetic pathway, depicted in Scheme 2, to obtain calixarbutin as target molecule. 

According to the Scheme 2, the synthetic strategy involves the grafting of four hydroxyl groups and glucosyl moieties at the upper and the lower rim of the calix[4]arene scaffold, respectively. The grafting of hydroxyl groups on the upper rim involved the conversion of tetra acetyl groups to tetra acetoxy groups via Baeyer-Villiger oxidation by perbenzoic acid on the lower rim of protected calix[4]arene **2**. This reaction required prolonged times in the dark, with occasional shaking at room temperature for completion. It was followed by the basic hydrolysis of tetra-acetoxy groups to tetra hydroxyl groups in the presence dioxane as solvent under reflux condition for 2 h. The compound **2** was synthetized by the S_N_2 reaction of calix[4]arene **1 **with α-acetobromoglucose in the presence of sodium iodide and potassium carbonate as a weak base in dry acetone under reflux condition for 6 h (13, 15). The structures of the all compounds were well characterized by NMR and FAB-MS spectra. Due to the zone of carbons’ chemical shifts (~37 ppm, ^13^C-NMR) of methylene bridges in the calixarene structures and the singlet pattern for protons of the methylene bridges in their ^1^H-NMR spectra, it can be concluded that all synthetic compounds have 1,3-alt conformation (18). The anomeric protons of compounds **2**,**3**, and **4** were assigned to the doublet at about 4.3 ppm with J_1,2 _~7 Hz, confirming the β-configuration. It confirms the S_N_2 mechanism for nucleophilic attack of calixarene to anomeric carbon of the sugars with configuration inversion. 

**Figure 1:**
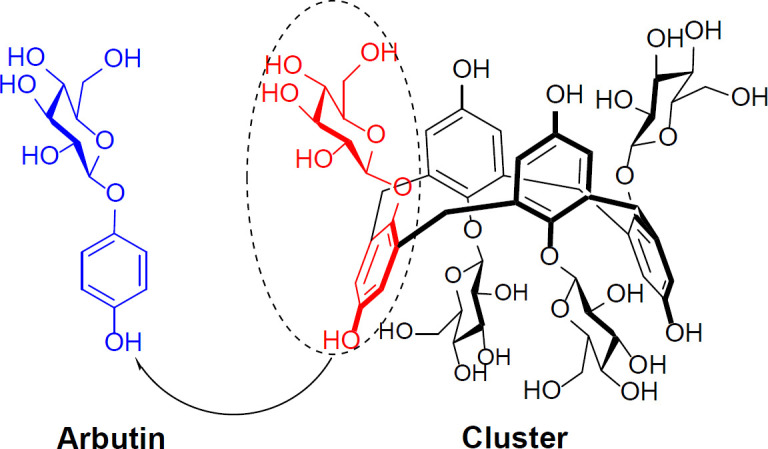
Structural comparison of arbutin with its corresponding cluster

According to Figure 1, the present chalice-shaped cluster is the first example of calixarene/arbutin hybrid and arbutin can be considered as ¼ of the synthetic cyclic tetramer.

*In-vitro biological evaluations*

One of the problems with using new drug structures in living systems is their poor aqueous solubility. Hence, due to the presence of four glycosyl units in the structure of calixarbutin, it can be high soluble in water. Therefore, the aqueous solubility of calixarbutin in comparison to arbutin was tested at 25 °C. To this purpose, they were slowly added to a specified volume of water and the addition was continued until saturation was reached. The results of this test and the other *in-vitro* biological evaluations for calixarbutin and arbutin are summarized in Table 1. According to these results, the water solubility of the synthesized cluster is about 8-fold more than of arbutin aqueous solubility.

In order to evaluate the potentially amplified biological activity of cluster, it has been compared with its corresponding phenolic monomer as a reference drug in the inhibition of the human melanoma cells’ growth and tyrosinase activity. 

Typically, in *in-vitro* tyrosinase inhibition assays, due to commercial availability, mushroom tyrosinase is used as a model of human tyrosinase to determine the values of dose-dependent inhibitory effect (IC_50_) of the test compounds by spectrophotometry study in phosphate buffer (2, 3). In anti-tyrosinase assay, IC_50_ refers to the concentration of a substance that inhibits a standard response by 50% of the enzyme activity. If the enzyme is completely inhibited, no dopachrome will be produced in response to L-DOPA addition to the reaction mixture. In other words, the catalytic activity of tyrosinase enzyme is directly related to the amount of produced dopachrome in the reaction mixture. Dopachrome is a red color intermediate in the process of melanin biosynthesis, and its amount is determined by measuring the absorbance at 492 nm by UV-Vis spectrophotometer (16).

According to Table 1, the results of mushroom tyrosinase assay based on DOPA-chrome method indicated that calixarbutin with lower IC_50_ values is about 6-fold more potent than its monomer arbutin in the inhibition of tyrosinase activity. The enhanced anti-tyrosinase activity of calixarbutin is maybe attributed to clustering effect of four impacted arbutin units. In addition, based on the structure of the active site of the tyrosinase (2, 3), it can be concluded that the enhanced inhibitory activity of calixarbutin with respect to its’ monomer can be due to its’ improved interaction with the enzyme central domain via the possibility of establishing more hydrogen bonds of the various hydroxyl groups on calixarbutin with amino-acides of the active site.

In addition, to determine the half maximal inhibitory growth concentration (IC_50_) values and the *in-vitro* cytotoxicity of calixarbutin in comparison to arbutin as a reference drug, they were evaluated against A375 human malignant melanoma cell line. The cells were cultured in a special medium (8) and then, the calixarbutin and arbutin were individually pre-dissolved in water and DMSO as stock solutions, respectively. The cells were exposed to a range of 1–1000 μg/mL of test compounds as working solutions. To avoid DMSO toxicity, the concentration of DMSO was less than 0.1% (v/v) in all experiments. The colorimetric assay of 3-(4,5-dimethylthiazol-2-yl)2,5-diphenyltetrazolium bromide (MTT) was used to establish cell viability by measurement of the absorbance of the resulting dye at 450 nm. The results of the cytotoxic activity, based on IC_50_ values, are summarized in Table 1. According to these results, calixarbutin with lower IC_50_ values, is about 27-fold more cytotoxic than arbutin against A375 cells. It can be attributed to the presence of four impacted arbutin arms in its structure and their synergistic effects.

**Table 1:** *In-vitro* bioactivities of calixarbutin and arbutin

**Compd.**	**Aqueous solubility** (mg/mL, 25 °C)	**Anti-tyrosinase activity** (IC_50_, μM)	**Anti-melanoma activity** (IC_50_, μg/mL)
Arbutin	38 ± 2	168 ± 7	988 ± 24
Calixarbutin	309 ± 13	26 ± 1	37 ± 2

## Conclusion

In summary, the present work describes a low-cost drug discovery research for the synthesis of the first calixarene-based cluster (cyclic tetramer in chaliced shape) of arbutin as a novel water-soluble melanogenesis inhibitor with improved anti-tyrosinase (6-fold) effect and amplified melanoma inhibitory (27-fold) activity in comparison to its phenolic monomer, as reference drug. In fact, arbutin can be considered as ¼ of the chalice-shaped cyclic tetramer. Compared to monomer, cluster with improved potencies in inhibition of tyrosinase function and A375 human melanoma cells is more “selective” water-soluble anti-tyrosinase agent with high antiproliferative effects on melanoma.

## References

[1] Nasuhi Pur F (2016). Calixdrugs: calixarene-based clusters of established therapeutic drug agents. Mol. Divers.

[2] Pillaiyar T, Manickam M, Namasivayam V (2017). Skin whitening agents: medicinal chemistry perspective of tyrosinase inhibitors. J. Enzyme Inhib. Med. Chem..

[3] Pillaiyar T, Namasivayam V, Manickam M, Jung SH (2018). Inhibitors of Melanogenesis: An Updated Review. J. Med. Chem..

[4] Zhu W, J Gao J (2008). The use of botanical extracts as topical skin-lightening agents for the improvement of skin pigmentation disorders. J. Investig. Dermatol. Symp. Proc..

[5] Kang MJ, Ha HW, Kim GH, Lee SK, Ahn YT, Kim DH, Jeong HG, Jeong TC (2012). Role of metabolism by intestinal bacteria in arbutin-induced suppression of lymphoproliferative response in-vitro. Biomol. Ther.

[6] Hori I, Nihei K, Kubo I (2004). Structural criteria for depigmenting mechanism of arbutin. Phytother. Res.

[7] Sugimoto K, Nishimura T, Nomura K, Sugimoto K, Kuriki T (2004). Inhibitory effects of alpha-arbutin on melanin synthesis in cultured human melanoma cells and a three-dimensional human skin model. Biol. Pharm. Bull.

[8] Nasuhi Pur F, Akbari Dilmaghani K (2014). Calixplatin: novel potential anticancer agent based on the platinum complex with functionalized calixarene. J. Coord. Chem.

[9] Nasuhi Pur F, Akbari Dilmaghani K (2014). Calixpenams: synthesis, characterization, and biological evaluation of penicillins V and X clustered by calixarene scaffold. Turk. J. Chem.

[10] Nasuhi Pur F, Akbari Dilmaghani K (2014). Calixcephems: clustered cephalosporins analogous to calixpenams as novel potential anti-MRSA agents. Turk. J. Chem.

[11] Nasuhi Pur F, Akbari Dilmaghani K (2015). Calixtyrosol: a novel calixarene based potent radical scavenger. Iran. J. Pharm. Res.

[12] Nasuhi Pur F, Akbari Dilmaghani K (2016). New antiradical clusters synthesized using the first green Biginelli reactions of calix[4]Arene. Pharm. Chem. J.

[13] Delnavaz Shahr A, Nasuhi Pur F, Akbari Dilmaghani K (2019). Calixapap: calixarene-based cluster of acetaminophen as a novel antiradical agent. Iran. J. Pharm. Res.

[14] Nasuhi Pur F, Delnavaz Shahr A, Akbari Dilmaghani K (2019). Calixmexitil: calixarene-based cluster of mexiletine with amplified anti-myotonic activity as a novel use-dependent sodium channel blocker. Iran. J. Pharm. Res.

[15] Redemann CE, Niemann C (1942). 2, 3, 4, 6-Tetraacetyl-α-d-gluco-pyranosyl bromide. Org. Synth.

[16] Song YM, Ha YM, Kim JA, Chung KW, Uehara Y, Lee KJ, Chun P, Byun Y, Chung HY, Moon HR (2012). Synthesis of novel azo-resveratrol, azo-oxyresveratrol and their derivatives as potent tyrosinase inhibitors. Bioorg. Med. Chem. Lett.

[17] Nihei K, Kubo I (2003). Identification of oxidation product of arbutin in mushroom tyrosinase assay system. Bioorg. Med. Chem. Lett.

[18] Jaime C, de Mendoza J, Prados P, Nieto PM, Sanchez C (1991). Carbon-13 NMR chemical shifts A single rule to determine the conformation of calix[4]arenes. J. Org. Chem..

